# Effects of bacteria-enzyme co-fermented Chinese herbal medicine on growth performance, apparent nutrient digestibility, meat quality, and immune function in broilers

**DOI:** 10.3389/fvets.2025.1676951

**Published:** 2025-09-25

**Authors:** Peng Ding, Xiong Wang, Sai Jiang, Minxi Li, Xi He, Yanmei Peng

**Affiliations:** ^1^Institute of Innovative Traditional Chinese Medications, Hunan Academy of Chinese Medicine, Changsha, China; ^2^FuRong Laboratory, Changsha, China; ^3^College of Animal Science and Technology, Hunan Agricultural University, Changsha, China; ^4^Hunan Baodong Agriculture and Animal Husbandry Co., Ltd., Changsha, China

**Keywords:** bacteria-enzyme co-fermented, Chinese herbal medicine, broilers, growth performance, apparent nutrient digestibility, immune function

## Abstract

This experiment aimed to investigate the effects of bacteria-enzyme co-fermented Chinese herbal medicine on growth performance, apparent nutrient digestibility, meat quality, and immune function in broilers. Four hundred one-day-old, uniformly healthy Arbor Acres broiler chicks were randomly divided into 5 groups with 8 replicates of 10 chicks each. Using a single-factor randomized design, the control group received a basal diet, the unfermented herbs group received the basal diet supplemented with 1% unfermented Chinese herbal medicine, and three treatment groups received the basal diet supplemented with 0.5, 1, and 1.5% bacteria-enzyme co-fermented Chinese herbal medicine, respectively. The feeding trial lasted 42 days. Upon completion, three broilers were randomly selected from each replicate for a 4-day metabolism trial to determine apparent nutrient digestibility. Results showed that compared to the control group, the final body weight and average daily gain were significantly increased (*p* < 0.05), and the feed-to-gain ratio was significantly decreased (*p* < 0.05) in the groups supplemented with 1 and 1.5% co-fermented herbs. The apparent digestibility of dry matter, crude ash, and gross energy was significantly higher (*p* < 0.05) in the 1 and 1.5% co-fermented herbs groups than in the control group. All three co-fermented herbs supplementation groups exhibited significantly higher apparent digestibility of crude protein, ether extract, and crude fiber compared to the control group (*p* < 0.05). Breast muscle drip loss and shear force were significantly reduced (*p* < 0.05) in the 1 and 1.5% co-fermented herbs groups compared to the control, with no significant difference between these two groups (*p* > 0.05). All herbs-supplemented groups showed significantly higher serum IgA, IgG, and IL-2 levels and significantly lower IL-1β levels than the control group (*p* < 0.05). In conclusion, supplementing Arbor Acres broiler diets with bacteria-enzyme co-fermented Chinese herbal medicine effectively enhanced growth performance and apparent nutrient digestibility, improved meat quality, and boosted immune function. Comprehensive consideration suggests that the recommended inclusion level of bacteria-enzyme co-fermented Chinese herbal medicine in Arbor Acres broiler diets under this experimental condition is 1%.

## Introduction

Under the context of a complete ban on antibiotics in feed, Chinese herbal medicine has garnered widespread attention in intensive livestock and poultry farming due to its advantages of being environmentally friendly, safe, low in toxicity and side effects, and posing minimal risks of drug resistance (1, 2). China boasts abundant herbal resources, with nearly 12,000 medicinal plant species currently available as potential bases for herbal feed additives. Through rational formulation and application, these herbs demonstrate multiple biological benefits in livestock and poultry production (3–5). In large-scale broiler farming, high stocking density per unit area frequently leads to common challenges such as respiratory diseases, heat stress, and bacterial enteritis (6, 7). Studies indicate that active components from *Coptis chinensis* (huanglian), *Forsythia suspensa* (lianqiao), and *Isatis indigotica* (banlangen) exhibit broad-spectrum antibacterial effects against pathogens prevalent in broiler farming, including *Salmonella*, *Escherichia coli*, *Staphylococcus*, and *Streptococcus hemolyticus*. These components show positive effects in preventing bacterial enteritis and mycoplasma infections in broilers (8–10), while also influencing meat quality (11). The combination of *Fritillaria cirrhosa* (chuanbeimu) and *Forsythia suspensa* demonstrates efficacy in dispelling wind-heat, alleviating heat stress in intensive farming systems, and reducing respiratory infections in flocks (12, 13). Polysaccharides derived from *Codonopsis pilosula* (dangshen) and *Citrus reticulata pericarp* (chenpi) enhance immune cell activity and improve specific immune functions (14, 15). However, certain herbs face limitations in widespread application due to poor palatability caused by anti-nutritional factors, which can be mitigated through fermentation processing (16, 17). Current fermentation techniques primarily involve probiotics and enzymes, yet single fermentation approaches often suffer from inconsistent outcomes and multiple constraints. The synergistic use of probiotics and enzymes has proven effective in improving fermentation efficiency, enhancing outcomes, and shortening fermentation cycles (18–21). This study aims to investigate the effects of applying a probiotic-enzyme synergistically fermented herbal formulation to *Arbor Acres* broiler diets, focusing on growth performance, apparent nutrient digestibility, meat quality, and immune function, thereby providing insights for optimizing herbal applications.

## Materials and methods

### Sample preparation

The raw materials of Chinese herbal medicine used in this trial were purchased from *Hunan Haodonglin Pharmaceutical* Co., Ltd. The formulation consisted of 20% *Coptis chinensis*, 15% *Isatis indigotica*, 15% *Forsythia suspensa*, 15% *Citri Reticulatae Pericarpium*, 15% *Codonopsis pilosula*, 15% *Citrus aurantium*, and 5% *Fritillaria cirrhosa*. The mixed herbs were pulverized and sieved through an 80-mesh sieve for later use. The bacteria-enzyme co-fermentation process was conducted at a local biotechnology company using *Bacillus coagulans*, *Aspergillus niger*, and *Bacillus licheniformis* as starter cultures, with an inoculation dose of 2 × 10^7^ CFU/g for each strain. After adding 0.5% composite enzymes, solid-state fermentation was performed at 37 °C for 7 days, followed by low-temperature air drying.

### Animal management

Hunan Agricultural University Animal Ethics Committee (Changsha, China) reviewed and approved all experimental protocols. A total of 400 one-day-old, healthy Arbor Acres broilers with uniform body conditions were randomly divided into 5 groups, each containing 8 replicates of 10 birds. The experiment followed a single-factor randomized design: the control group was fed a basal diet; the unfermented herbal group received the basal diet supplemented with 1% unfermented Chinese herbal medicine; and the three experimental groups were fed the basal diet supplemented with 0.5, 1, and 1.5% bacteria-enzyme co-fermented Chinese herbal medicine, respectively. The basal diet was formulated according to the nutritional requirements of Arbor Acres broilers, and its composition and nutrient levels are presented in Table 1. All birds had free access to feed and water throughout the 42-day trial period. Upon completion of the feeding trial, 3 birds were randomly selected from each replicate for a 4-day metabolic trial to determine the apparent digestibility of nutrients.

**Table 1:** Basal diet composition and nutrient levels (air-dried basis).

Item	1–21 d	22–42 d
Ingredient (%)		
Corn	59.50	66.25
Soybean meal	25.80	18.42
Cottonseed meal	4.50	6.50
Corn gluten meal	3.80	2.80
Soybean oil	2.50	2.50
Limestone	1.50	1.50
Salt	0.50	0.50
*L*-Lysine HCl	0.45	0.30
*DL*-Methionine	0.35	0.15
*L*-Threonine	0.10	0.08
Premix①	1.00	1.00
Total	100.00	100.00
Nutrient level②		
Metabolizable energy (MJ/kg)	12.89	13.12
Crude protein	20.91	19.95
Lysine	0.89	0.84
Methionine	0.31	0.28
Calcium	1.01	1.11
Total phosphorus	0.72	0.75

① Premix provides per kg of diet: vitamin A 8700 IU, vitamin D_3_ 1800 IU, vitamin E 38 mg, vitamin K_3_ 0.38 mg, vitamin B_1_ 1.80 mg, vitamin B_2_ 12 mg, vitamin B_6_ 3.0 mg, niacin 50 mg, pantothenic acid 8 mg, folic acid 1.20 mg, Cu (copper) 26.50 mg, Fe (iron) 48.00 mg, Mn (manganese) 40.00 mg, Zn (zinc) 38.00 mg, I (iodine) 0.75 mg, Se (selenium) 0.18 mg. ② Nutrient levels: crude protein, calcium, and total phosphorus are measured values; other nutrient levels are calculated values.

Broilers were reared in a single-tier cage system with 10 birds per cage. Feeding management and environmental temperature control followed standard farm protocols, including regular cleaning and disinfection of the poultry house. At the end of the trial, one bird was randomly selected from each replicate group. Blood samples (5–10 mL) were collected from the brachial vein using vacuum blood collection tubes. The tubes were tilted and left to stand for 30 min, then centrifuged at 1500 × *g* for 15 min to separate serum. The serum was aliquoted into 1.5 mL microcentrifuge tubes and stored at −20 °C for subsequent immune parameter analysis. After slaughter, the left pectoral muscle was collected to measure pH, meat color, drip loss, and shear force. Prior to the initiation of the metabolism trial, the excrement trays beneath the cages were cleaned and lined with oiled paper. Fresh fecal samples (approximately 50 g) were collected daily at 18:00. Feathers, feed residues, and other contaminants were removed from the feces using forceps. Subsequently, 5 mL of 10% dilute hydrochloric acid was added to fix nitrogen. Samples were then stored at −20 °C. Finally, feces collected over four consecutive days were pooled according to biological replicates, dried at 65 °C, pulverized, and sealed in containers for subsequent analysis. At the start of the feeding trial, one-day-old broilers were weighed per replicate. Weekly feed consumption and residual feed quantities were recorded per replicate throughout the trial. On the final day of the trial, birds were reweighed per replicate to calculate average daily gain (ADG), average daily feed intake (ADFI), and feed-to-gain ratio (F/G).

### Apparent nutrient digestibility in broilers

The apparent digestibility of nutrients was determined using acid-insoluble ash (AIA) in the diet and feces as an endogenous indicator. The calculation formula is as follows:


$$X\%=(1-\frac{S}{F}\times \frac{Y}{B})\times 100$$

Where:


X%=Apparent digestibility ofaspecific nutrient

S=AIAcontent in the diet(%)

F=AIAcontent in feces(%)

Y=Nutrient content in feces(%)

B=Nutrient content in the diet(%)

### Meat quality assessment in broilers

pH: Measured using a pH meter (*pH-STAR*, *Matthäus*, *Germany*) on the same muscle sample at 45 min and 24 h post-slaughter. Meat color: Evaluated using a colorimeter (*Minolta CM-2600d/2500d*, *Minolta Camera Co*., *Ltd*., *Japan*) on the same sample at 45 min and 24 h. Parameters recorded: lightness (L*), redness (a*), and yellowness (b*). Drip loss: The left pectoral muscle was cut into strips (1 cm × 1 cm × 2 cm), weighed, suspended with iron wire/cotton thread in a disposable plastic cup (ensuring no contact with the cup), placed inverted in a ziplock bag, and stored at 4 °C for 24 h. After reweighing, drip loss was calculated as:


$$\text{Driploss}(\%)=\frac{\text{Initial weight}-\text{Final weight}}{\text{Initial weight}}\times 100$$

Shear force: Pectoral muscle samples were cut into strips (~1 cm thick) and measured using a tenderness meter.

### Serum immune parameters

Serum immunoglobulin A (IgA, *Kit No. H108-1-2*), immunoglobulin G (IgG, *Kit No. H106-1-1*), immunoglobulin M (IgM, *Kit No. E025-1-1*), interleukin-1β (IL-1β, *Kit No. H002-1-2*), and interleukin-2 (IL-2, *Kit No. H003-1-1*) were analyzed using commercial kits according to the manufacturers’ protocols. Specifically: IgA, IgG, IL-1β, and IL-2 were measured by enzyme-linked immunosorbent assay (ELISA). IgM was determined via immunoturbidimetric assay. All kits were sourced from *Nanjing Jiancheng Bioengineering Institute* (*China*).

### Statistical analysis

Final results are presented as mean ± standard deviation (SD). Data were initially processed using box plots in SPSS 22.0 and assessment of variance homogeneity. Subsequently, one-way analysis of variance (ANOVA) was performed for all experimental groups. For datasets showing significant differences, multiple comparisons were conducted using Duncan’s multiple range test. Linear and quadratic regression analyses were conducted on the control group and the three co-fermented herbs supplementation groups to determine whether there was a dose-effect relationship of the supplementation levels. Statistical significance was defined at *p* < 0.05.

## Results

### Growth performance

As evidenced by the Arbor Acres broiler growth performance data in Table 2, supplementation with 1 and 1.5% bacteria-enzyme co-fermented herbs significantly increased final body weight and ADG (*p* < 0.05) while significantly reducing the F/G compared to the control group (*p* < 0.05). No significant differences were observed in any growth parameters between the 1 and 1.5% co-fermented herbs groups (*p* > 0.05). Conversely, neither the 1% unfermented herbs group nor the 0.5% co-fermented herbs group showed statistically significant differences in growth performance relative to the control group (*p* > 0.05). Dietary inclusion levels of co-fermented herbs exhibited linear correlations with final body weight, ADG, and ADFI in broilers (*p* < 0.05).

**Table 2:** Effects of bacteria-enzyme co-fermented Chinese herbal medicine on growth performance of broilers.

Item	Control group	1% Unfermented herbs group	Co-fermented herbs supplementation			*p*-value^*^		
			0.5%	1.0%	1.5%	*A*	*L*	*Q*
Initial weight (g)	50.52 ± 0.32	50.49 ± 0.35	50.48 ± 0.37	50.51 ± 0.28	50.51 ± 0.30	0.280	0.125	0.336
Final weight (g)	2339.84 ± 33.47^b^	2366.70 ± 40.26^b^	2458.25 ± 38.66^ab^	2589.73 ± 39.12^a^	2602.31 ± 37.49^a^	0.039	0.032	0.264
ADG (g)	54.51 ± 1.04^b^	55.15 ± 1.08^b^	57.33 ± 1.11^b^	60.46 ± 1.09^a^	60.76 ± 1.12^a^	0.022	0.015	0.591
ADFI (g)	91.57 ± 4.24	92.10 ± 3.85	93.44 ± 2.99	93.71 ± 4.37	94.79 ± 4.10	0.307	0.024	0.157
F/G	1.68 ± 0.09^a^	1.67 ± 0.10^a^	1.63 ± 0.11^ab^	1.55 ± 0.10^b^	1.56 ± 0.08^b^	0.012	0.215	0.660

^*^A denotes the p-value from one-way ANOVA among groups, while L and Q represent the p-values for linear and quadratic regression analyses between the control group and the three co-fermented herbs supplementation groups, respectively. Within a row, values with different superscript letters (a, b, ab) differ significantly (*p* < 0.05). Values with the same letter or no letter indicate no significant difference (*p* > 0.05). The same applies to the tables below.

### Apparent digestibility of nutrients

Table 3 demonstrates that dietary supplementation with 1 and 1.5% bacteria-enzyme co-fermented herbs significantly increased (*p* < 0.05) the apparent digestibility of dry matter, ash, and gross energy in AA broilers compared to the control group. Furthermore, all three co-fermented herbs supplementation groups (0.5, 1, and 1.5%) exhibited significantly higher (*p* < 0.05) apparent digestibility of crude protein, ether extract, and crude fiber than the control. However, no significant differences (*p* > 0.05) were observed in nutrient digestibility between the 1 and 1.5% co-fermented herbs groups. Dietary inclusion levels of co-fermented herbs exhibited linear correlations (*p* < 0.05) with apparent digestibility of dry matter, ether extract, crude fiber, crude ash, and gross energy, whereas a tendency toward quadratic correlation (*p*  > 0.05) was observed for crude protein digestibility in broilers.

**Table 3:** Effects of bacteria-enzyme co-fermented Chinese herbal medicine on apparent nutrient digestibility (%) in broilers.

Item	Control group	1% Unfermented herbs group	Co-fermented herbs supplementation			*p*-value^*^		
			0.5%	1.0%	1.5%	*A*	*L*	*Q*
Dry matter (%)	88.71 ± 1.33^b^	88.66 ± 1.47^b^	90.03 ± 1.53^ab^	92.83 ± 1.72^a^	92.80 ± 1.66^a^	0.013	0.027	0.513
Crude protein (%)	50.17 ± 5.26^c^	52.50 ± 7.35^c^	60.34 ± 6.42^b^	65.99 ± 5.20^a^	65.64 ± 7.11^a^	0.002	0.158	0.071
Ether extract (%)	60.65 ± 6.36^b^	61.02 ± 7.19^b^	65.75 ± 7.33^a^	66.12 ± 6.54^a^	66.11 ± 8.15^a^	0.022	0.034	0.194
Crude fiber (%)	76.86 ± 3.64^b^	76.05 ± 5.25^b^	80.38 ± 5.11^a^	80.32 ± 5.37^a^	81.50 ± 4.28^a^	0.004	0.011	0.452
Ash (%)	68.92 ± 9.18^b^	68.37 ± 9.66^a^	75.58 ± 10.42^ab^	81.15 ± 10.27^a^	80.32 ± 9.80^a^	0.013	0.048	0.127
Gross energy (%)	78.36 ± 6.33^b^	78.47 ± 5.54^b^	83.22 ± 6.28^ab^	88.30 ± 7.10^a^	88.17 ± 6.18^a^	0.018	0.029	0.184

### Meat quality

As evidenced by Table 4, supplementation with 1 and 1.5% bacteria-enzyme co-fermented herbs significantly reduced drip loss and shear force in the pectoral muscle of AA broilers compared to the control group (*p* < 0.05), with no significant difference observed between these two treatment groups (*p* > 0.05). Conversely, no statistically significant differences were detected among all groups for pH values or meat color parameters (L*, a*, b*) at either 45 min or 24 h post-slaughter (*p* > 0.05). Dietary inclusion levels of co-fermented herbs exhibited linear correlations with pectoral muscle drip loss and shear force in broilers (*p* < 0.05).

**Table 4:** Effects of bacteria-enzyme co-fermented Chinese herbal medicine on meat quality of broilers.

Item		Control group	1% Unfermented herbs group	Co-fermented herbs supplementation			*P*-value^*^		
				0.5%	1.0%	1.5%	*A*	*L*	*Q*
pH	45 min	6.12 ± 0.18	6.11 ± 0.19	6.12 ± 0.20	6.11 ± 0.18	6.12 ± 0.17	0.124	0.135	0.886
	24 h	5.53 ± 0.17	5.52 ± 0.22	5.53 ± 0.24	5.56 ± 0.25	5.52 ± 0.23	0.645	0.227	0.126
Meat color 45 min	L*	42.35 ± 2.54	42.35 ± 2.65	42.48 ± 2.82	42.63 ± 2.33	42.50 ± 3.14	0.880	0.392	0.163
	a*	5.62 ± 0.54	5.58 ± 0.30	5.80 ± 0.45	5.77 ± 0.29	5.69 ± 0.33	0.103	0.289	0.247
	b*	1.51 ± 0.11	1.48 ± 0.08	1.50 ± 0.09	1.48 ± 0.09	1.50 ± 0.10	0.349	0.534	0.364
Meat color 24 h	L*	47.01 ± 3.28	47.33 ± 3.19	47.24 ± 2.39	47.68 ± 4.10	47.33 ± 3.27	0.432	0.654	0.702
	a*	12.35 ± 1.92	12.20 ± 2.24	13.63 ± 1.99	13.49 ± 2.17	13.11 ± 2.08	0.842	0.553	0.781
	b*	2.63 ± 0.12	2.73 ± 0.13	2.76 ± 0.14	2.80 ± 0.18	2.91 ± 0.17	0.333	0.697	0.448
Drip loss (%)		2.44 ± 0.11^a^	2.32 ± 0.09^a^	2.21 ± 0.12^ab^	2.01 ± 0.11^b^	1.97 ± 0.09^b^	0.029	0.017	0.288
Shear force (N)		44.66 ± 7.59^a^	44.32 ± 8.45^a^	44.47 ± 7.64^a^	40.22 ± 8.37^b^	40.18 ± 8.26^b^	0.031	0.025	0.376

### Immune function

As shown in Table 5, all Chinese herbal supplementation groups exhibited significantly higher serum IgA, IgG, and IL-2 levels (*p* < 0.05) but significantly lower IL-1β levels (*p* < 0.05) compared to the control group, while no significant differences (*p* > 0.05) were observed in IgM levels among all groups. Dietary inclusion levels of co-fermented herbs exhibited linear correlations with serum IgA and IgG levels in broilers (*p* < 0.05).

**Table 5:** Effects of bacteria-enzyme co-fermented herbs on immune function of broilers.

Item	Control group	1% Unfermented herbs group	Co-fermented herbs supplementation			*p*-value^*^		
			0.5%	1.0%	1.5%	*A*	*L*	*Q*
IgA (g/L)	1.44 ± 0.05^b^	1.67 ± 0.07^a^	1.68 ± 0.04^a^	1.72 ± 0.03^a^	1.73 ± 0.07^a^	0.037	0.025	0.316
IgG (g/L)	3.11 ± 0.65^b^	4.47 ± 0.63^a^	4.49 ± 0.40^a^	4.51 ± 0.44^a^	4.62 ± 0.56^a^	0.014	0.015	0.157
IgM (g/L)	0.64 ± 0.14	0.65 ± 0.18	0.64 ± 0.22	0.68 ± 0.17	0.62 ± 0.19	0.391	0.258	0.518
IL-1β (ng/L)	34.11 ± 2.99^a^	31.06 ± 3.12^b^	30.89 ± 3.18^b^	30.76 ± 2.72^b^	30.88 ± 2.17^b^	0.026	0.207	0.361
IL-2 (ng/L)	227.04 ± 15.38^b^	243.56 ± 17.06^a^	244.27 ± 16.51^a^	246.09 ± 12.44^a^	245.21 ± 12.76^a^	0.018	0.368	0.669

## Discussion

The enhancement of growth performance in intensive livestock operations is pivotal for economic viability. Bacteria-enzyme co-fermentation demonstrates superior efficacy in degrading fibrous materials through synergistic interactions between probiotics and enzymes (17, 22), particularly in processing herbal substrates where it effectively neutralizes anti-nutritional factors and toxins, thereby improving feed palatability (23). Notably, this technology outperforms single-component fermentation by accelerating substrate decomposition and reducing processing time (24). These advantages align with Chen et al.’s findings (25), where co-fermented herbs enhanced growth performance and gut microbiota diversity in weaned piglets. Mechanistically, the *Citri Reticulatae Pericarpium–Citrus aurantium* synergy promotes digestive health via qi regulation and spleen fortification (26, 27), mitigating heat stress-induced bloat and hypophagia. Concurrently, *Codonopsis pilosula* counters performance decline by replenishing qi and blood (28). Critically, our data substantiate these mechanisms: 1–1.5% co-fermented herb supplementation significantly elevated final weight, ADG, and feed efficiency in AA broilers, establishing a robust growth performance response.

Enhancing feed digestibility is pivotal for reducing costs and improving economic returns in livestock production. Fermentation processing disrupts the plant cell walls of herbal materials, facilitating the release of bioactive compounds (29) while reducing toxic constituents such as alkaloids, thereby mitigating gastrointestinal damage. Concurrently, microbial metabolites synergize with herbal components in the digestive tract (19), collectively enhancing nutrient utilization. Notably, *Coptis chinensis* and *Forsythia suspensa* classified as cold-natured herbs in Traditional Chinese Medicine (TCM) undergo thermal modulation during fermentation. Probiotic-derived thermogenic metabolites neutralize their cold properties (30), reducing enteric irritation in broilers and improving nutrient digestibility, which broadens their applicability in animal production. Critically, ester compounds generated via bacteria-enzyme co-fermentation mask bitter notes in feedstuffs, improving palatability. The concomitant organic acids not only inhibit mycotoxin formation but also promote nutrient absorption (31). Our results validate these mechanisms: co-fermented herbs significantly elevated apparent digestibility of conventional nutrients, indicating enhanced digestive function in broilers.

Broiler meat quality is intricately linked to genetic strain, dietary composition, rearing environment, and slaughter protocols, where its enhancement critically extends shelf life and consumer appeal. Various phytochemicals found in herbs—including flavonoids, saponins, and phenolics—can modulate lipid metabolism, thereby influencing muscle flavor and texture (31–33). For instance, berberine from *Coptis chinensis* suppresses lipogenic enzymes, which reduces abdominal and subcutaneous fat deposition while promoting fatty acid oxidation (34). Additionally, *Codonopsis* polysaccharides have been shown to enhance protein deposition and elevate the levels of flavor-enhancing amino acids, such as glutamate and aspartate (14, 15). Meanwhile, hesperidin in *Citri Reticulatae Pericarpium* upregulating antioxidant enzymes to mitigate heat stress and preserve post-slaughter color and water-holding capacity (35), where water-holding capacity dictates shelf stability. Crucially, our data demonstrate that 1–1.5% co-fermented herbs significantly improved water-holding capacity and reduced shear force in broilers, confirming their efficacy in enhancing meat quality.

Enhanced immune competence in intensively reared broilers constitutes the foundation for sustained growth performance. Research indicates that polysaccharides from *Codonopsis pilosula* critically augment macrophage activity and nonspecific immunity (36, 37). Within the herbal formulation, *Forsythia suspensa* activates immune organs to accelerate antibody production (38), synergizing with the anti-inflammatory and antioxidant properties of *Coptis chinensis* and *Isatis indigotica* to mitigate inflammatory responses while elevating antioxidative capacity (39, 40). Berberine from *Coptis chinensis* and bioactive compounds in *Isatis indigotica* inhibit viral replication (41, 42), exhibiting broad-spectrum antibacterial efficacy against common bacterial enteritis and mycoplasma infections (43, 44). *Fritillaria cirrhosa*, clinically employed for its lung-moistening and mucolytic effects, cooperates with *Forsythia suspensa* to prevent respiratory infections in dense populations (45). Critically, serum immunoglobulin and cytokine profiles reflect immune status—our results confirm that herbal supplementation beneficially modulated these parameters in broilers, demonstrating enhanced immunocompetence attributable to the integrated phytocomplex.

In summary, dietary supplementation with bacteria-enzyme co-fermented Chinese herbal medicine effectively enhances growth performance and nutrient digestibility while improving meat quality and immune function in Arbor Acres broilers. Under the experimental conditions, the optimal inclusion level is determined at 1%.

## Data Availability

The original contributions presented in the study are included in the article/supplementary material, further inquiries can be directed to the corresponding author/s.
